# Autophagy Regulates Ferroptosis-Mediated Diabetic Liver Injury by Modulating the Degradation of ACSL4

**DOI:** 10.1155/jdr/7146054

**Published:** 2024-12-24

**Authors:** Liangxiu Wu, Weicheng Lai, Lanlan Li, Sen Yang, Fengjuan Li, Chen Yang, Xiaobing Gong, Liangyan Wu

**Affiliations:** ^1^Department of Gastroenterology, The First Affiliated Hospital of Jinan University, Guangzhou, China; ^2^Department of Gastroenterology, The People's Hospital of Hezhou, Hezhou, China; ^3^Department of Cardiology, Nanjing BenQ Medical Center, The Affiliated BenQ Hospital of Nanjing Medical University, Nanjing, China; ^4^Department of Endocrinology, Sun Yat-sen Memorial Hospital, Sun Yat-sen University, Guangzhou, China; ^5^Department of Cardiovascular Medicine, The First Affiliated Hospital of Jinan University, Guangzhou, China; ^6^Department of Endocrinology and Metabolism, Zhuhai People's Hospital (Zhuhai Clinical Medical College of Jinan University), Zhuhai, China; ^7^Department of Endocrinology and Metabolism, The First Affiliated Hospital of Jinan University, Guangzhou, China

**Keywords:** acyl-CoA synthetase long-chain family member 4, autophagy, diabetes mellitus, ferroptosis, liver injury

## Abstract

**Background:** Diabetic liver injury is a serious complication due to the lack of effective treatments and the unclear pathogenesis. Ferroptosis, a form of cell death involving reactive oxygen species (ROS)-dependent lipid peroxidation (LPO), is closely linked to autophagy and diabetic complications. Therefore, this study is aimed at investigating the role of autophagy in regulating ferroptosis by modulating the degradation of acyl-CoA synthetase long-chain family member 4 (ACSL4) in diabetic hepatocytes and its potential impact on diabetic liver injury.

**Methods:** Initially, ferroptosis and autophagy were assessed in liver tissues from streptozotocin-induced diabetic rats and in palmitic acid (PA)–treated LO2 cells. Subsequently, the study focused on elucidating the regulatory role of autophagy in mediating ferroptosis through the modulation of ACSL4 expression in PA-treated LO2 cells.

**Results:** The results demonstrated that ACSL4-mediated ferroptosis and inhibition of autophagy were observed in diabetic hepatocytes in vivo and in PA-treated LO2 cells. Additionally, the ferroptosis inhibitor was able to mitigate the PA-induced cell death in LO2 cells. Mechanistically, the stability and expression level of the ACSL4 protein were upregulated and primarily degraded via the autophagy-lysosome pathway in PA-treated LO2 cells. The use of the autophagy inhibitor 3-methyladenine (3-MA) and the inducer rapamycin further demonstrated that autophagy regulated ferroptosis by mediating ACSL4 degradation, highlighting its critical role in diabetic liver injury.

**Conclusions:** These results elucidate the roles of ferroptosis, autophagy, and their interactions in the pathogenesis of diabetic liver injury, offering potential therapeutic targets. Furthermore, they shed light on the pathogenesis of ferroptosis and other diabetic complications.

## 1. Introduction

Diabetes mellitus (DM) is a globally occurring and chronic noncommunicable disease characterized by the dysregulation of carbohydrate, protein, and fat metabolism [1]. DM seriously threatens the liver, as it can lead to conditions such as nonalcoholic steatohepatitis, nonalcoholic fatty liver disease, and even hepatocellular carcinoma [2]. Diabetic liver injury is a common complication that develops following DM, attributed to hyperglycemia and/or hyperlipidemia [3]. However, the specific mechanisms underlying the development of diabetic liver injury remain unknown. The liver plays a crucial role in lipid metabolism; the accumulation of excessive amounts of lipids greatly contributes to lipotoxicity and high levels of reactive oxygen species (ROS) [4]. These changes eventually damage hepatocytes and even induce apoptosis [5]. Besides apoptosis, ROS accumulation is a crucial characteristic and mechanism underlying ferroptosis. However, whether hyperlipidemia-mediated lipotoxicity and ROS accumulation can promote ferroptosis in diabetic hepatocytes; thus, inducing diabetic liver injury should be further investigated.

Ferroptosis, proposed by Dixon et al. in 2012, represents a form of iron-dependent cell death distinct from apoptosis [6]. The occurrence of ferroptosis depends on overwhelming lipid peroxidation (LPO) produced enzymatically or nonenzymatically, which could be reduced by a lipid repair enzyme called glutathione peroxidase 4 (GPX4) [7]. The enzyme acyl-CoA synthetase long-chain family member 4 (ACSL4) plays a key role in the synthesis of arachidonic acid-PE and adrenaline-PE and facilitates the conversion of polyunsaturated fatty acids (PUFA) in phospholipids into lipid peroxides. This enzymatic activity is a part of the execution phase of ferroptosis [8, 9]. Doll et al. have found that the ferroptosis inducer RSL3, which causes ferroptosis through the GPX4 pathway, cannot do so after the ACSL4 gene is knocked out [8]. Therefore, it can be asserted that ACSL4 is not only a sensitive indicator of ferroptosis but also a key contributor to ferroptosis [8, 10]. Studies have indicated that ferroptosis induction by ACSL4 plays a vital role in aggravating the effects of diabetes and its associated complications [11, 12], such as diabetic cardiomyopathy [13, 14], diabetic nephropathy [15], and diabetic retinopathy [16]. Hence, further research is needed to determine whether the upregulation of ACSL4 plays a role in diabetic liver damage through the induction of ferroptosis.

Ferroptosis is a type of cell death that depends on autophagy, as it requires the autophagic machinery to promote oxidative damage [17, 18]. Autophagy not only acts as a catabolic process for degrading cytoplasmic proteins [19] but also decreases oxidative stress and inflammation [20]. Due to its multifaceted role, autophagy is associated with various diseases, including cancer, inflammatory conditions, neurodegenerative disorders, and metabolic diseases [21]. The impairment of autophagy may occur because of diabetes and its complications [22]. Autophagic flux recovery can improve insulin resistance [23], diabetic cardiomyopathy [24], and diabetic nephropathy [25]. Some researchers also have suggested that autophagy dysfunction may cause diabetic liver injury [26], and liver injury in Type 2 diabetes can be alleviated due to the promotion of AMPK/mTOR-mediated autophagy [27]. Ferroptosis is closely associated with autophagy. Previous studies have shown that autophagy can mediate ferroptosis through several mechanisms, including NCOA4-mediated ferritin autophagy enhancing ferritin degradation [28], BECN1-mediated suppression of the system XC [29], RAB7A-dependent lipid degradation via lipophagy, STAT3-induced lysosomal membrane permeabilization [30], and HSP90-related chaperone-mediated autophagy [31]. Hence, whether autophagy can regulate ferroptosis through other ways needs further investigation. In eukaryotic cells, protein degradation primarily occurs through the autophagy-lysosome cascade and the ubiquitin-proteasome network [32, 33]. However, the initiator of the ferroptosis ACSL4 degradation pathway needs to be determined. Therefore, we investigated whether autophagy induces ferroptosis by mediating ACSL4 degradation in diabetic liver damage.

We found that ACSL4-mediated ferroptosis increased and that autophagy was inhibited in the livers of streptozotocin (STZ)-induced diabetic rats, as well as in palmitic acid (PA)–treated LO2 cells. Autophagy also played a role in ferroptosis by regulating the expression of ACSL4. In summary, we elucidated a novel mechanism by which autophagy contributes to ferroptosis and exacerbates diabetic liver damage by inhibiting the degradation of ACSL4. These findings may provide insights into novel therapeutic strategies for treating diabetic liver injury by targeting ferroptosis or autophagy.

## 2. Materials and Methods

### 2.1. The Development of Animal Models for Diabetes

We obtained healthy male Sprague–Dawley (SD) rats from the Department of Laboratory Animals Center in Guangdong Province, China. All animal experimental procedures were approved by the Institutional Animal Care and Use Committee at Sun Yat-sen University (SYSU-IACUC-2023–000470) and were conducted strictly following the approved protocol. Initially, the SD rats were allowed to freely consume standard chow and water and were acclimated to the experimental environment for at least 1 week. Then, six SD rats were induced into a diabetic state through intraperitoneal injection of 60 mg/kg STZ (Sigma, St. Louis, MO, United States) following overnight fasting. Four rats in the control group received an equivalent volume of sodium citrate buffer via intraperitoneal injection. Five rats with fasting plasma glucose levels exceeding 300 mg/dL 7 days post-STZ injection were considered to be in a diabetic state. At the termination of the experiment after 12 weeks, the rats underwent anesthesia with secobarbital (50 mg/kg) and were sacrificed by cervical dislocation. Serum was isolated from blood samples and then immediately stored at −80°C for subsequent biochemical analyses. After liver tissues were dissected, one portion was fixed in paraffin or frozen in an optimal cutting temperature compound, and the rest was preserved in liquid nitrogen for subsequent analyses.

### 2.2. Liver Function and Biochemistry Measurement

The serum was separated from blood samples by centrifugation at 1000 × *g* for 10 min and was used to conduct standard liver function and biochemistry analyses. An automated biochemical analyzer (Siemens, Germany) was used to quantify triglyceride (TG), total cholesterol (TC), aspartate aminotransferase (AST), and alanine transaminase (ALT) levels.

### 2.3. LPO Assessment

Superoxide dismutase (SOD) activity and malondialdehyde (MDA) content were evaluated to quantify LPO in liver tissues and serum. The MDA content and the SOD activity were quantified using commercial assay kits (A003-1 and A001-1, Jiancheng Bioengineering Institute, Nanjing, China), following the manufacturer's instructions.

### 2.4. Measurement of Ferrous Iron (Fe^2+^) Content

The concentration of Fe^2+^ in serum and liver tissues was assessed using an iron assay kit (A039-2, Jiancheng Bioengineering Institute, Nanjing, China), following the manufacturer's instructions. In total, iron in 10 *μ*L of serum or 10 mg of tissue homogenate was determined by measuring the absorbance at 593 nm and comparing them with a standard curve of known concentrations.

### 2.5. Measurement of ROS

The frozen liver specimens were thawed at room temperature. A spontaneous fluorescence quenching reagent was applied to the liver tissue sections and incubated for 5 min. Following this, a ROS staining solution (D7008, Sigma, Saint Louis, MO, United States) was added, and the mixture was incubated at 37°C for 30 min in the dark. After the samples were thoroughly rinsed with phosphate-buffered saline (PBS), the liver tissue sections were stained with 4⁣′,6-diamidino-2-phenylindole (DAPI) solution for 10 min at room temperature. After washing thrice in a warm PBS, the samples were observed under a fluorescence microscope.

### 2.6. Oil Red O Staining

The LO2 cells and liver tissues were fixed at room temperature with paraformaldehyde (4%) for 30 min and washed thrice with PBS. Next, they were stained with Oil Red O solution (G1016, Servicebio, Wuhan, China) for 10 min, rinsed in water, and examined under a light microscope (Nikon, Melville, NY, United States).

### 2.7. Transmission Electron Microscopy (TEM)

After the 12-week-long experiment was terminated, the rats were euthanized and their livers were harvested. The livers were rinsed in PBS and fixed using 2.5% glutaraldehyde. Subsequently, liver tissues were cut into thin sections (50 *μ*m thick), with specific regions treated with 1% osmium tetroxide for 1 h. The samples were then dehydrated through a graded ethanol series and embedded in epoxy resin. The polymerization process lasted 24 h at 80°C. Ultrathin slices (100 nm) were observed using a JEM2000EX transmission electron microscope (JEOL, Tokyo, Japan) after being sliced and stained with lead citrate and uranyl acetate.

### 2.8. Cell Culture

Human hepatocyte LO2 cells were obtained from the China Center for Type Culture Collection (CCTCC, Wuhan, China) and cultured in Dulbecco's modified Eagle's medium (Gibco, United States) supplemented with 10% fetal bovine serum and 100 U/mL penicillin and streptomycin at 5% CO_2_. LO2 cells were treated with different concentrations of high glucose (HG) for specific durations to replicate an HG environment. Another set of LO2 cells was treated with different concentrations of PA (P101058, Aladdin, Shanghai, China) for 24 h to establish the hyperlipidemia model.

For the ACSL4 knockdown and overexpression experiments in LO2 cells, ACSL4 overexpression and control vectors were synthesized by WZ Biosciences. The structure of the ACSL4 overexpression plasmid is shown in Supplementary Information 3. LO2 cells were seeded in 12-well plates. According to the manufacturer's instructions, 1 *μ*g of plasmids were transfected into LO2 cells using Lipofectamine 3000 reagent (#L3000015, Thermo Fisher, United States). For PA intervention while simultaneously knocking down ACSL4, small interfering RNA (siRNA) designed and synthesized by RiboBio was used. Cells were again seeded in 12-well plates, and 100 pmol of siRNA was transfected using Lipofectamine RNAiMAX (#13778150, Invitrogen, United States), following the manufacturer's guidelines. The siRNA sequence was shown below: si-ACSL4-#1 GAGCGATTTGAAATTCCAA and si-ACSL4-#2 ACAGCATGCAATCAGTAGA.

### 2.9. Cycloheximide (CHX), MG132, and Bafilomycin A1 (Baf A1) Treatment

After 24-h PA treatment, LO2 cells were incubated with 100 *μ*g/mL CHX for 0, 6, 12, or 16 h for subsequent analysis. MG132 is an inhibitor of the proteasomal pathway of protein degradation, whereas Baf A1 is an inhibitor of the lysosomal pathway. The LO2 cells were treated with 20 *μ*M MG132 and 20 nM Baf A1 for 8 h, respectively, after which, they were collected and processed for protein extraction and Western blotting analysis.

### 2.10. Cell Counting Kit-8 (CCK-8) Assay

Cell viability was evaluated through a CCK-8 assay (CW Biotech, Beijing, China). First, suspended LO2 cells were incubated with different concentrations of PA for 24 h or were exposed to different concentrations of HG medium for 48 or 72 h. After treatment, cells in each well were treated with 10 *μ*L of CCK-8. Then, the absorbance values were measured at 450 nm using a Multiscan Spectrum (Thermo Fisher Scientific, MA, United States).

### 2.11. Lactate Dehydrogenase (LDH) Activity Assay

The LDH activity was determined using a standard LDH cytotoxicity assay kit (Beyotime Biotechnology, Shanghai, China), following the manufacturer's instructions. The supernatant was collected from the cell culture, and the primary reactant mixture was added. After incubating the cells for 30 min, the absorbance was measured at 490 nm using a spectrophotometer.

### 2.12. BODIPY 581/591 C11 Assay

The LPO content in LO2 cells was evaluated using the BODIPY581/591 C11 LPO sensor (D3861, Thermo Fisher, Wilmington, DE, United States). The LO2 cells were cultured in six-well plates, harvested by the trypsin-ethylenediaminetetraacetic acid (EDTA) digestion method, and washed with PBS. For BODIPY-C11 staining, cells were suspended in 500 *μ*L of serum-free medium containing 1 *μ*M BODIPY 581/591 C11 and incubated in a tissue incubator at 37°C for 40 min. After the cells were washed, they were resuspended in 200 *μ*L of PBS. Generally, the fluorescence emission peak shifts from ~590 to ~510 nm as the PUFA butadienyl component in the dye oxidizes. This shift in the fluorescence peak was measured by flow cytometry (Beckman Coulter, Fullerton, CA, United States), following a standardized protocol.

### 2.13. Analysis of Cellular Apoptosis

The apoptosis of LO2 cells was analyzed using the Annexin V-fluorescein isothiocyanate (FITC) cell apoptosis kit (Beyotime, Shanghai, China). In total, 1 × 10^6^ cells were seeded in 12-well culture plates and treated. Following the manufacturer's instructions, the cells were resuspended in 500 *μ*L of binding buffer and stained for 15 min in the dark with 5 *μ*L of Annexin V-FITC and 10 *μ*L of propidium iodide (PI). Flow cytometry (Beckman Coulter, Fullerton, CA, United States) was performed to analyze apoptosis following established protocols.

### 2.14. GFP-LC3 Puncta Analysis

The LO2 cells were cultured in a tool focal plate, and autophagic flux was assessed using the Premo Autophagy Tandem Sensor mRFP-GFP-LC3 Kit (P36239, Thermo Fisher, Wilmington, DE, United States), following the manufacturer's instructions. The LO2 cells were transfected with mRFP-GFP-LC3 and incubated overnight, followed by treatment with PA or bovine serum albumin (BSA) for 24 h, as indicated. Next, the cells were treated for 10 min with paraformaldehyde for fixation and then washed with PBS. After the paraformaldehyde was removed, the LO2 cells were rinsed thrice with PBS at room temperature and kept in the dark. Confocal microscopy was performed to capture fluorescence images.

### 2.15. Real-Time Quantitative Polymerase Chain Reaction (RT-qPCR)

The levels of the ACSL4 gene were quantified by the RT-qPCR assay. Total RNA was extracted from rat livers and LO2 cells using TRIzol (Takara, Otsu, Shiga, Japan), following the manufacturer's instructions. The extracted RNA served as a template for cDNA synthesis. The RT-qPCR analysis was performed using a Light Cycler System and Light Cycler 480 SYBR Green Master Mix (Roche Diagnostics, Mannheim, Germany). The coding areas were amplified using the primers listed below: ACSL4 (human), forward: 5⁣′-ACTGGCCGACCTAAGGGAG-3⁣′, reverse: 5⁣′-GCCAAAGGCAAGTAGCCAATA-3⁣′; ACSL4 (rat), forward: 5⁣′-GATCCCAGGAGATTGACCTGT-3⁣′, reverse: 5⁣′-CTGGAGAAGGCAGTAACGGAA-3⁣′; *β*-actin (human), forward: 5⁣′-TGGAACGGTGAAGGTGACAG-3⁣′, reverse: 5⁣′-AACAACGCATCTCATATTTGGAA-3⁣′; *β*-actin (rat), forward: 5⁣′-CCCGCGAGTACAACCTTCTT-3⁣′, reverse: 5⁣′-CCACGATGGAGGGGAAGAC-3⁣′. Each value represents the average of at least three independent experiments.

### 2.16. Western Blotting Analysis

Total proteins were isolated from cells and liver tissues using radioimmunoprecipitation analysis (RIPA) lysis buffer (CW Biotech, Beijing, China) supplemented with protease and phosphatase inhibitors (CW Biotech, Beijing, China). The lysates were diluted with protein loading buffer (Solarbio, Wuhan, China) at a 1:5 ratio and heated at 100°C for 10 min. Equal volumes of proteins were then used to perform Western blotting analysis. A 12.5% sodium dodecyl sulfate-polyacrylamide gel was used to perform electrophoresis of the proteins, which were then transferred to polyvinylidene difluoride (PVDF) membranes (Merck-Millipore, Billerica, MA, United States). After transferring, the membranes were blocked with 5% skim milk for 1 h. Next, the samples were incubated with primary antibodies against ACSL4 (#ab155282, Abcam, Cambridge, United Kingdom), 4-hydroxynonenal (4HNE) (MAB3249, R&D Systems, Minneapolis, MN, United States), LC3-I/II (3868s, Thermo Fisher, Wilmington, DE, United States), p62 (#ab56416, Abcam, Cambridge, United Kingdom), and Beclin1 (#ab62557, Abcam, Cambridge, United Kingdom). Following this, the samples were incubated with secondary antibodies (Servicebio, Wuhan, China), and immunoreactivity was detected using an enhanced chemiluminescence reagent. The ImageJ software was used to calculate the band density.

### 2.17. Immunohistochemistry (IHC) and Hematoxylin and Eosin (H&E) Staining

First, liver tissues were fixed in 4% paraformaldehyde for 24 h, followed by paraffin embedding, and finally, they were cut into thin sections (4 mm thick) and mounted on glass slides. The sections were processed through several steps, including washing, dehydration with gradient ethanol, dewaxing, hydration, and blocking with 5% BSA at room temperature for 30 min, as per the methodology detailed in our previous study [34]. Then, the liver sections were incubated with primary antibodies against ACSL4, 4HNE, p62, and LC3 overnight at 4°C. After the liver sections were washed thrice in PBS (10 min each wash), they were treated with the relevant secondary antibodies for 1 h at room temperature. The coverslip was sealed with neutral glue, and the histopathological conditions were observed under an optical microscope. H&E staining of the liver tissue was performed using an H&E staining reagent (Servicebio, Wuhan, China) following standard protocols.

### 2.18. Statistical Analysis

All data were expressed as the mean ± standard deviation(Std Dev) based on a minimum of three independent experiments. All statistical analyses were performed using IBM SPSS Statistics 24.0. The differences between and among groups were determined by the two-tailed unpaired Student's *t*-test or one-way analysis of variance (ANOVA) with Bonferroni's test or Dunnett's test, as indicated in the corresponding figure legends. All differences among the experimental groups were considered statistically significant at *p* < 0.05.

## 3. Results

### 3.1. ACSL4-Mediated Ferroptosis Was Detected in the Liver of STZ-Induced Diabetic Rats

**Figure 1:**
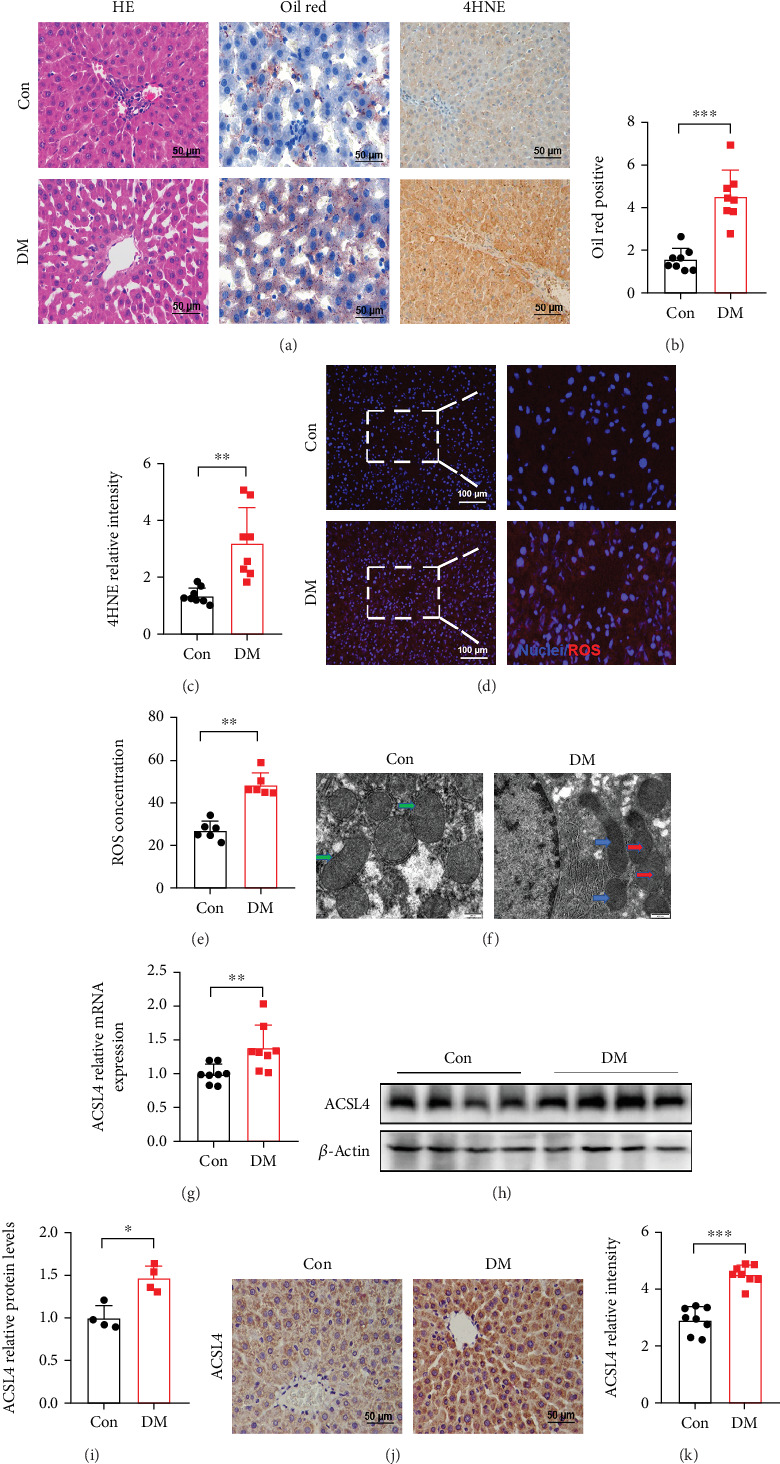
ACSL4-mediated ferroptosis was detected in STZ-induced diabetic rats. (a) Representative H&E staining, Oil Red O staining, and 4HNE immunohistochemical staining of the liver of rats; scale bar: 50 *μ*m. (b) The quantitative analysis of Oil Red O–stained cells. (c) Quantification of the immunohistochemical staining of 4HNE in each group. (d) Representative immunofluorescence images of ROS in each group; scale bar: 100 *μ*m. (e) Quantitative analysis of the fluorescence intensity of ROS. (f) The morphological changes in mitochondria in the hepatocytes of rats were detected by TEM. The green arrow indicates normal mitochondria, the blue arrow indicates outer mitochondrial membrane rupture, and the red arrows indicate the reduction or disappearance of mitochondrial cristae; scale bar: 500 nm. (g) RT-qPCR analysis of the expression of ACSL4 mRNA in the liver of rats. (h, i) Representative Western blots and quantitative densitometry analysis of the expression of the ACSL4 protein in the liver of rats. (j, k) Representative images and quantitative analysis of ACSL4 in the liver, as determined by IHC; scale bar: 50 *μ*m. All data are presented as the mean ± Std Dev; *n* = 4 per group; ⁣^∗^*p* < 0.05, ⁣^∗∗^*p* < 0.01, and ⁣^∗∗∗^*p* < 0.001 versus Con.

To explore whether ferroptosis is involved in diabetic liver injury, STZ-induced diabetic rats were established to explore this question. We confirmed that the diabetic model was successfully constructed by recording high blood glucose levels and a decrease in body weight post-STZ injection (Table 1). The rats in the model group also exhibited significantly higher levels of TG and TC compared to those in the control group, which indicated dyslipidemia in the diabetic rats (Table 1). After 12 weeks of STZ-induced diabetes, serum concentrations of ALT and AST were considerably higher in the diabetic rats than in the nondiabetic rats (Table 1). Pathological alterations in diabetic liver tissue were assessed via H&E staining. The hepatocytes in the diabetic group were disorganized and contained a large number of lipid droplets, whereas those in the control group were neatly arranged without any signs of steatosis (Figure 1(a)). Liver lipid deposition was assessed by Oil Red O staining, which confirmed our previous results. Lipid deposition was considerably greater in the diabetic group than in the control group (Figures 1(a) and 1(b)). These findings indicated that the diabetic rats experienced dyslipidemia, lipid deposition in the liver, and impaired liver function.

To assess the implication of ferroptosis in diabetic liver injury, we first evaluated the levels of LPO, Fe^2+^ concentration, and the expression of specific ferroptosis markers. We found the levels of MDA and Fe^2+^ in the serum and liver samples were significantly higher in the DM group than in the control group, indicating increased LPO in DM (Table 1). The level of SOD, an important antioxidant, exhibited the opposite trend, which indicated a reduction in the ability of SOD to remove ROS (Table 1). The degradation of lipid peroxides leads to the formation of 4HNE along with other compounds. The IHC-stained liver tissues in the DM group had a considerably greater abundance of 4HNE than those in the control group (Figures 1(a) and 1(c)). Additionally, red fluorescence in liver cells was stronger in the diabetic group than in the control group, suggesting higher ROS production in the liver cells of the diabetic group (Figures 1(d) and 1(e)). By analyzing the morphological characteristics of ferroptosis from TEM images, we found that the mitochondria in the hepatocytes of diabetic rats exhibited prominent morphological alterations, characterized by a reduction in mitochondrial size, a decrease in or disappearance of mitochondrial ridges, rupture of the mitochondrial outer membrane, and mitochondrial vacuolization (Figure 1(f)). ACSL4 is a key enzyme associated with the accumulation of lipid peroxides and plays a crucial role in ferroptosis. The results of RT-qPCR analysis showed that the level of expression of the ACSL4 mRNA was significantly higher in the DM group (Figure 1(g)). The results of the Western blotting and IHC analyses also showed that the level of the ACSL4 protein was significantly higher in the diabetic liver injury group (Figures 1(h), 1(i), 1(j), and 1(k)). To further validate our results, we examined the ACSL4 expression in the liver of the db/db mice. The results of Supporting Information 4 showed that the protein expression of ACSL4 was significantly increased in the liver of the db/db mice. Our findings suggested that ferroptosis occurs in diabetic liver injury and is associated with the upregulation of the initiator ACSL4.

### 3.2. Autophagy Was Inhibited in the Liver of STZ-Induced Diabetic Rats

**Figure 2:**
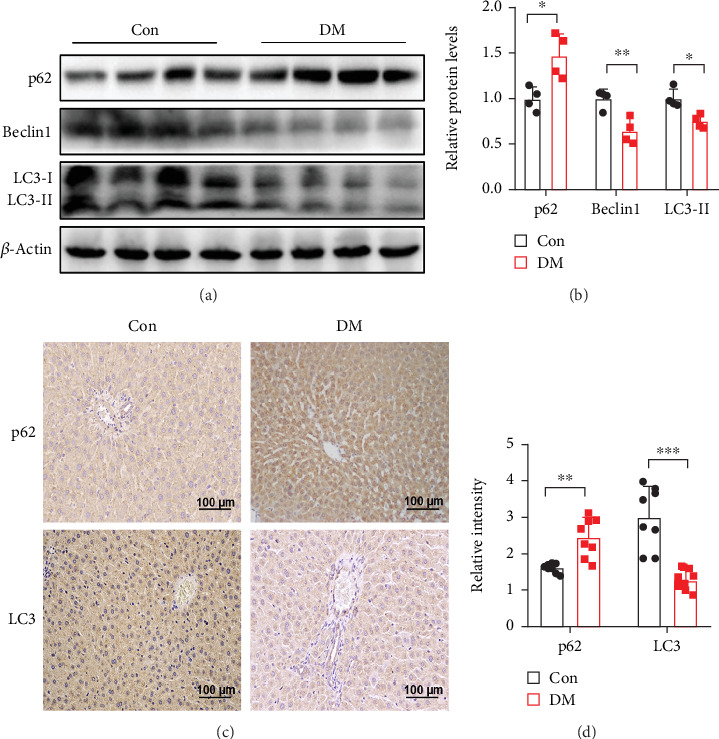
Autophagy was inhibited in the liver of STZ-induced diabetic rats. (a, b) Representative Western blots and quantitative densitometry analysis of LC3-II, Beclin1, and p62 protein expression in the liver of rats. (c, d) Representative images and quantitative analysis of LC3 and p62 in the liver of rats, as determined by IHC; scale bar: 100 *μ*m. All data are presented as the mean ± Std Dev; *n* = 4 per group; ⁣^∗^*p* < 0.05, ⁣^∗∗^*p* < 0.01, and ⁣^∗∗∗^*p* < 0.001 versus Con.

We evaluated autophagy-related proteins in the liver of STZ-induced diabetic rats. The conversion of the autophagy-related protein LC3 from its soluble form (LC3-I) to its lipid-bound form (LC3-II) acts as a marker of autophagy. This conversion can be identified by examining the accumulation of LC3-II or the formation of LC3-positive autophagosomes. p62 serves as a cargo adapter for the lysosomal degradation of ubiquitin, and its expression is inversely related to autophagy [35]. The results of Western blotting analysis showed that the level of p62 protein increased, coupled with a decrease in the levels of the proteins Beclin1 and LC3-II in the rats of the DM group compared to their corresponding levels in the control group (Figures 2(a) and 2(b)). Immunohistochemical staining confirmed these observations, demonstrating a reduction in the expression of LC3-II and an increase in the expression of p62 in the liver of STZ-induced diabetic rats (Figures 2(c) and 2(d)). These findings collectively indicated that autophagy was inhibited in the liver of diabetic rats.

### 3.3. Ferroptosis Inhibitor Attenuated Ferroptosis-Related Death in LO2 Cells Under PA Treatment

To investigate whether hepatocytes in the diabetic state underwent ferroptosis, LO2 cells were first exposed to HG. The results indicated that there was no discernible variation in the viability and cytotoxicity of LO2 cells cultured with different concentrations of HG for different durations (Supporting Information 1). The results of the LDH cytotoxicity assay showed that the percentage of LDH released was not significantly different among the groups (Supporting Information 1). The results of the Annexin V-FITC analysis also demonstrated that 33 mM HG did not significantly affect the apoptosis of LO2 cells (Supporting Information 1). Besides HG, hyperlipidemia is also an important feature of diabetes. Diabetics are more likely to suffer from hyperlipidemia than nondiabetics. The diabetic rats in our study showed signs of lipid metabolism disorders in the liver. PA is a high-fat inducer that induces steatohepatitis through lipid accumulation, insulin resistance, and chronic inflammatory reactions [36]. We administered PA to induce hyperlipidemia in vitro. The results of the CCK-8 assay showed that the viability of LO2 cells decreased with an increase in the concentration of PA after 24 h (Supporting Information 1). Based on these findings, we selected 300 *μ*M as the optimal concentration of PA for our subsequent experiments.

**Figure 3:**
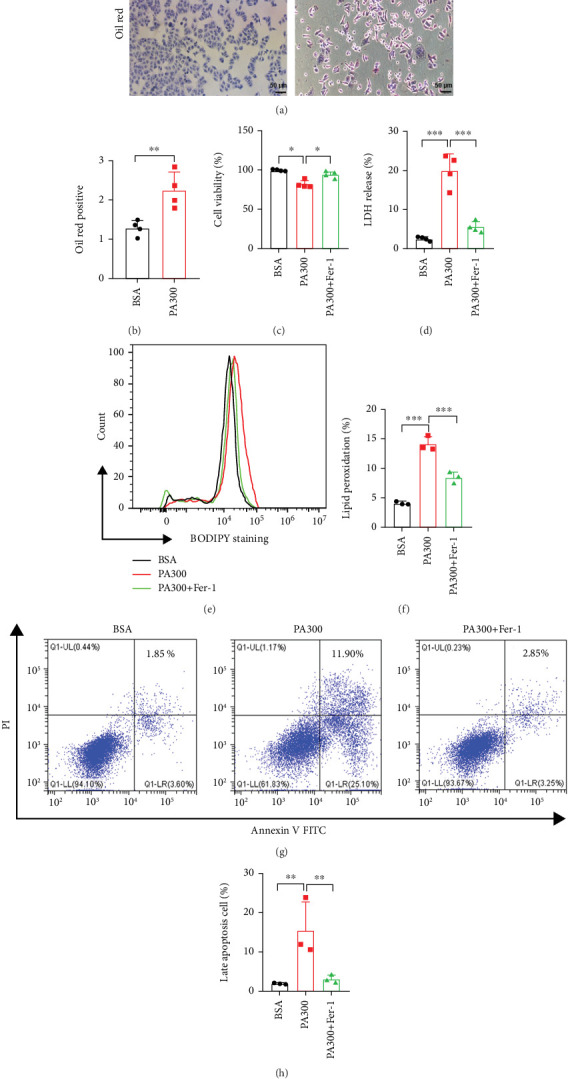
Ferroptosis inhibitor attenuated ferroptosis-related death in LO2 cells under PA treatment. (a) Representative Oil Red O staining of the LO2 cells cultured in 300 *μ*M PA for 24 h; scale bar: 100 *μ*m. (b) The quantitative analysis of Oil Red O–stained cells. (c) The viability of LO2 cells treated with PA or PA and Fer-1 was assessed. The cells were treated with PA (300 *μ*M) or PA (300 *μ*M) and Fer-1 (60 *μ*M) for 24 h, and then the viability of cells in each group was measured by the CCK-8 assay. (d) The level of the LDH released in LO2 cells treated with PA or PA and Fer-1 was measured using the LDH cytotoxicity assay kit. (e, f) The LO2 cells were stained with the LPO-specific dye BODIPY 581/591 C11, and LPO was detected by flow cytometry after the LO2 cells were treated with PA or PA and Fer-1. (g, h) LO2 cells were treated with PA or PA and Fer-1, stained with Annexin V-FITC and PI, and then cells that underwent apoptosis were quantified by flow cytometry; ⁣^∗^*p* < 0.05, ⁣^∗∗^*p* < 0.01, and ⁣^∗∗∗^*p* < 0.001 between the indicated groups.

**Figure 4:**
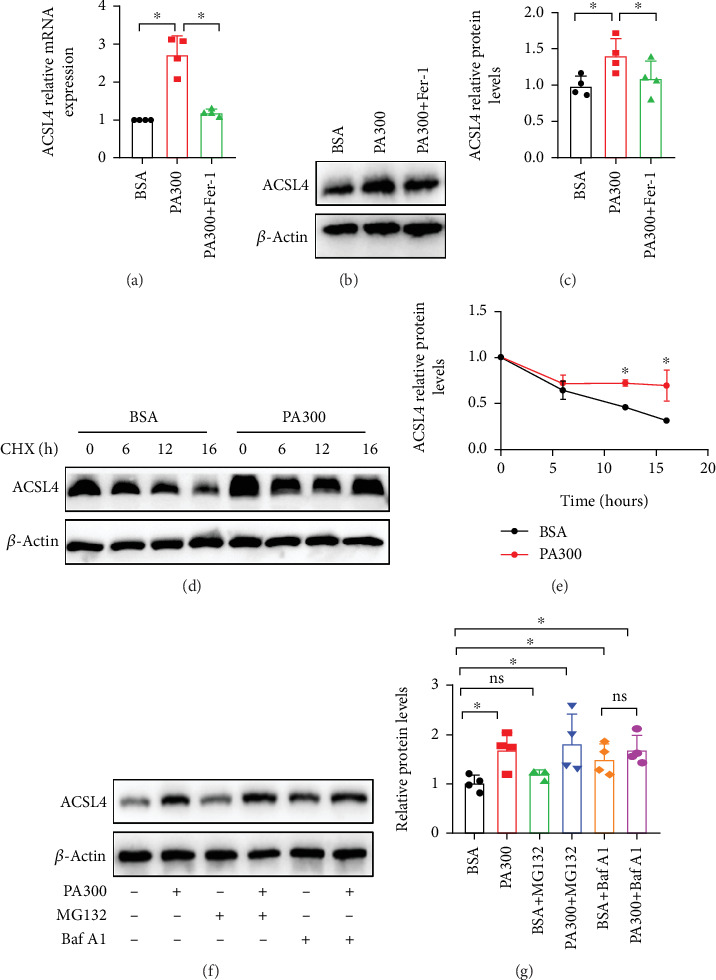
ACSL4 increased under PA treatment and was degraded through the autophagy-lysosomal pathway. (a) RT-qPCR analysis of the expression of ACSL4 mRNA in the LO2 cells treated with PA or PA and Fer-1. (b, c) Representative Western blots and quantitative densitometry analysis of ACSL4 protein expression in LO2 cells treated with PA or PA and Fer-1. (d, e) Protein stability of ACSL4 in LO2 cells cultured with PA, following a time course treatment with 100 *μ*g/mL CHX. (f, g) LO2 cells were treated with PA with or without MG132 (20 *μ*M) or Baf A1 (20 nM) for 8 h. Representative Western blots and quantitative densitometry analysis of the expression of the ACSL4 protein in LO2 cells. All data are presented as the mean ± Std Dev of at least three independent experiments; ⁣^∗^*p* < 0.05 between the indicated groups.

The LO2 cells that underwent Oil Red O staining showed intracellular lipid accumulation after treatment with PA for 24 h (Figures 3(a) and 3(b)). This finding confirmed that an in vitro hepatic steatosis model was established through PA treatment. We evaluated the cytotoxicity of the LO2 cells treated with PA and found that the proliferation of cells decreased significantly following treatment with 300 *μ*M PA, as determined by the CCK-8 assay (Figure 3(c)). The results of the LDH-cytotoxicity assay showed that the LO2 cells treated with PA experienced high cytotoxicity (Figure 3(d)). The results of C11-BODIPY staining revealed that the LPO levels were significantly higher in LO2 cells treated with PA (Figures 3(e) and 3(f)). The results of Annexin V-FITC analysis demonstrated an increase in the late apoptosis of LO2 cells treated with PA (Figures 3(g) and 3(h)). The results of RT-qPCR and Western blotting analyses showed that the expression of ACSL4, a key enzyme involved in triggering ferroptosis, was substantially upregulated in the PA-treated group (Figures 4(a), 4(b), and 4(c)). Thus, these results suggested hyperlipidemia is a critical factor contributing to hepatocyte ferroptosis in diabetes.

Ferrostatin-1 (Fer-1), a specific inhibitor of ferroptosis, was further used to determine whether the cell death induced by PA in LO2 cells was associated with ferroptosis. We found that the Fer-1 group experienced increased cell viability, reduced cytotoxicity, diminished LPO, and decreased apoptosis compared to the PA-treated group (Figures 3(c), 3(d), 3(e), 3(f), 3(g), and 3(h)). The Fer-1 group also exhibited lower ACSL4 mRNA and protein levels than the PA-treated group (Figures 4(a), 4(b), and 4(c)). These results indicated that the cell death induced by PA in LO2 cells is consistent with ferroptosis, and its progression can be mitigated by Fer-1.

**Figure 5:**
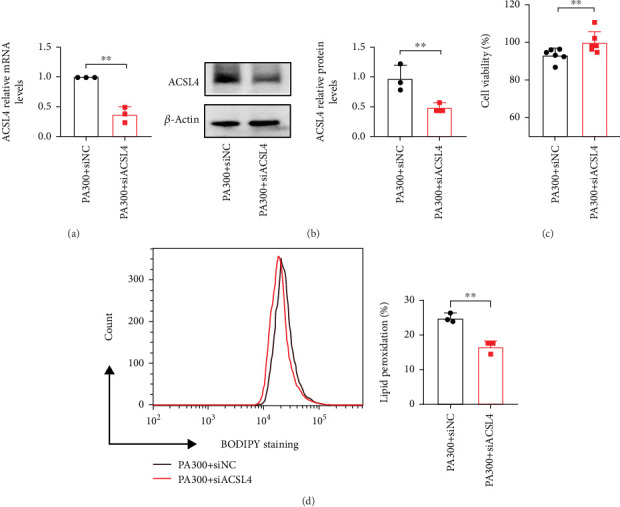
Downregulation of ACSL4 reversed PA-induced ferroptosis in LO2 cells. (a) qPCR analysis of ACSL4 mRNA expression in PA300+siNC or PA300 + siACSL4 LO2 cells. (b) Immunoblotting analysis of ACSL4 protein expression in PA300+siNC or PA300+siACSL4 LO2 cells. (c) Cell viability of PA-stimulated LO2 cells transfected with control and siACSL4 knockdown RNA. (d) Rate of LPO of PA-stimulated LO2 cells transfected with control and siACSL4 knockdown RNA. Bars represent the mean ± Std Dev; ⁣^∗∗^*p* < 0.01 between the indicated groups.

And to verify the role of ACSL4 in vitro, we performed the following experiments. First, LO2 cells were transfected with the ACSL4 overexpression and control plasmids (Supporting Information 2). ACSL4 overexpression increased cell death (Supporting Information 2), decreased cell viability (Supporting Information 2), and increased cellular LPO levels (Supporting Information 2). In summary, these results suggested that ACSL4 can mediate ferroptosis in LO2 cells. To investigate whether ACSL4 inhibition prevents PA-induced ferroptosis in LO2 cells, we knocked down ACSL4 expression with siRNA in PA-intervening LO2 cells. As the results suggested, PA-induced elevation of ACSL4 was also reversed by siACSL4 (Figures 5(a) and 5(b)). And ACSL4 knockdown improved the negative effects of cell viability and decreased cellular LPO levels in PA-stimulated LO2 cells (Figures 5(c) and 5(d)).

### 3.4. ACSL4 Can Be Degraded Through the Autophagy-Lysosomal Pathway

To elucidate the molecular mechanism underlying the significant increase in the level of ACSL4 protein in the hepatocytes of diabetic rats and PA-stimulated LO2 cells, we used the de novo protein synthesis inhibitor CHX. ACSL4 was more stable in PA-treated LO2 cells after 100 *μ*g/mL CHX was administered (Figures 4(d) and 5(e)), indicating that PA interfered with the degradation of ACSL4. In eukaryotic cells, proteins are degraded mainly by the ubiquitin-proteasome pathway and the autophagy-lysosomal pathway. To find which of the two pathways mediated ACSL4 degradation, Baf A1 and MG132 were used to elucidate the mechanism underlying the regulation of ACSL4 stability. After treatment with 20 nM Baf A1 (lysosome inhibitor) or 20 *μ*M MG132 (proteasome inhibitor) for 8 h, only Baf A1 enhanced the stability of ACSL4 in the BSA group. Moreover, the combination of Baf A1 with PA treatment could not induce a stronger inhibitory effect on ACSL4 degradation (Figures 4(f) and 4(g)). These results suggested that PA can increase the stability of ACSL4 through the autophagy-lysosomal pathway rather than the ubiquitin-proteasome pathway.

### 3.5. Inhibition of Autophagic Flux in PA-Treated LO2 Cells

**Figure 6:**
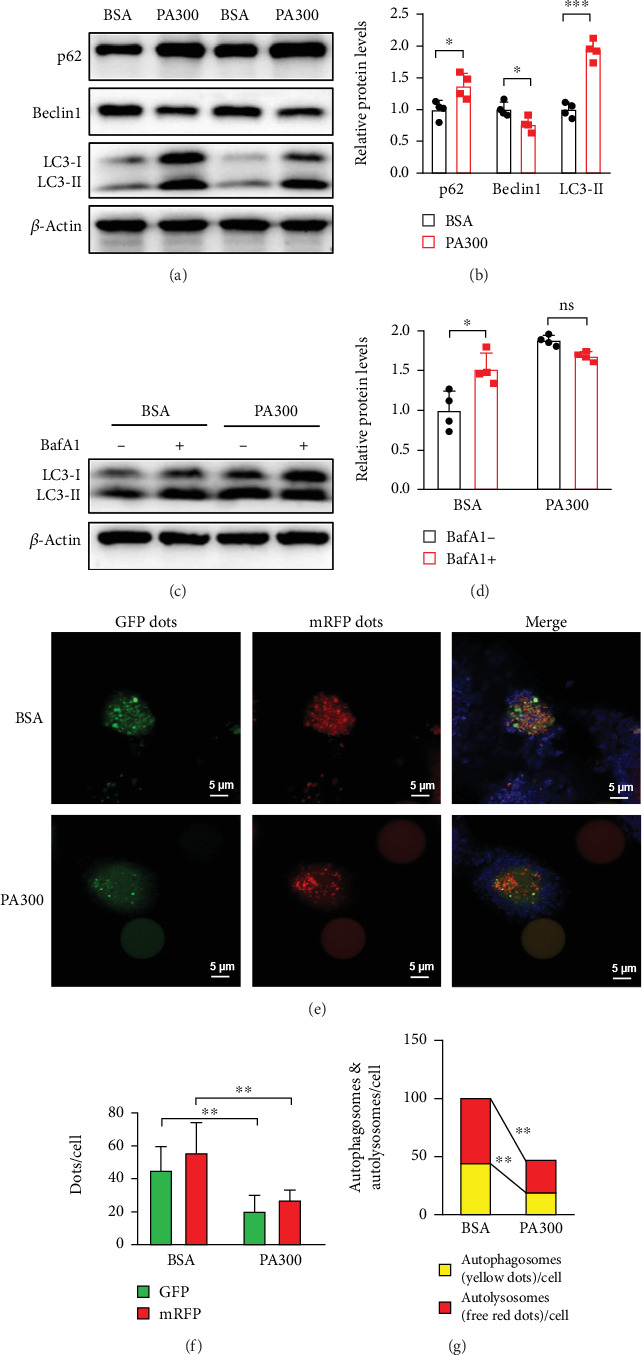
Inhibition of autophagic flux in PA-treated LO2 cells. (a, b) Representative Western blots and quantitative densitometry analysis of the expression of LC3-II, Beclin1, and p62 proteins in the LO2 cells exposed to PA for 24 h. (c, d) The LO2 cells were treated with PA for 24 h in the presence or absence of 20 nM Baf A1 during the last 2 h of coculture. Representative Western blots and quantitative densitometry analysis of the expression of the LC3-II protein in LO2 cells. (e) Representative images of LO2 cells infected with mRFP-GFP-LC3 and exposed to PA; Green: GFP puncta; Red: mRFP puncta. (f) GFP dots and mRFP dots were counted in each cell. (g) Semiquantitative analysis of autophagosomes (yellow puncta in merged images) and autolysosomes (red puncta in merged images). All data are presented as the mean ± Std Dev of at least three separate experiments; ns: nonsignificant; ⁣^∗^*p* < 0.05, ⁣^∗∗^*p* < 0.01, and ⁣^∗∗∗^*p* < 0.001 between the indicated groups.

To investigate the effect of PA on autophagy in LO2 cells, we estimated the level of the proteins LC3, Beclin1, and p62. After treatment with 300 *μ*M PA for 24 h, the levels of p62 and LC3-II increased considerably, accompanied by a decrease in the level of Beclin1 (Figures 6(a) and 6(b)). These changes in protein levels suggested compromised autophagic flux. To assess PA-induced autophagic flux, we used Baf A1, a pharmacological autophagy inhibitor. Baf A1 prevents autophagosomes from fusing with lysosomes, thereby reducing lysosomal enzyme activity.

We found that the expression of LC3-II increased in the presence of Baf A1 in the control group; however, Baf A1 did not affect the expression of LC3-II in PA-treated cells (Figures 6(c) and 6(d)), suggesting impaired autophagic flux. Since the RFP signal in the mRFP-GFP-LC3 fluorescent puncta assay is more stable than the GFP signal under acidic conditions, autophagy flux can be determined by counting the number of yellow puncta (autophagosomes) and red puncta (autolysosomes). A lower number of red and yellow spots in PA-treated LO2 cells indicated that fewer autophagosomes and autolysosomes were formed, indicating that autophagic flux was inhibited (Figures 6(e), 6(f), and 6(g)). These results suggested that PA can inhibit autophagic flux in LO2 cells.

### 3.6. Autophagy May Participate in Ferroptosis by Regulating the Expression of ACSL4

**Figure 7:**
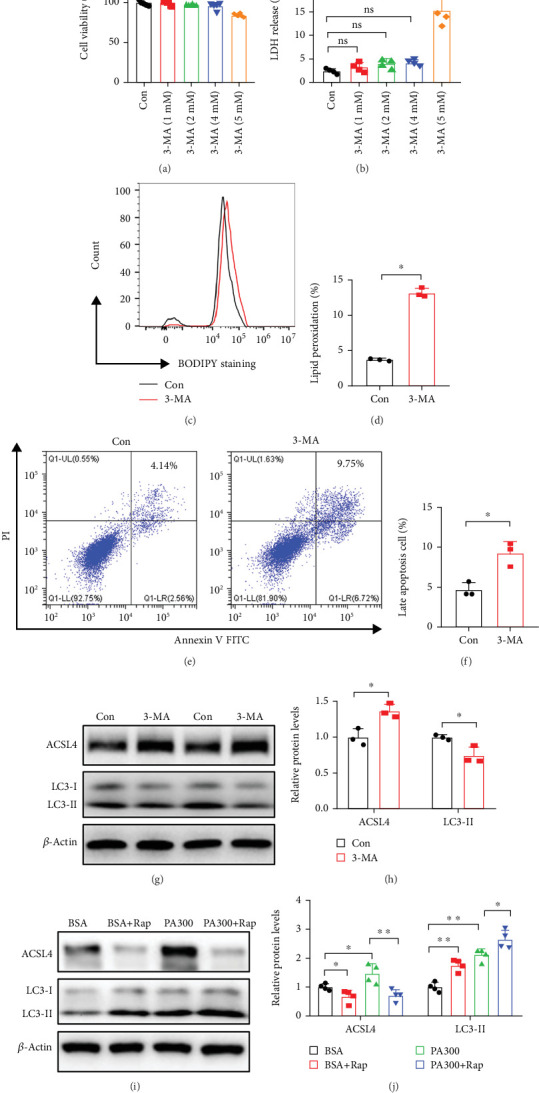
Autophagy may be involved in ferroptosis by regulating the expression of ACSL4. (a) CCK-8 assay was performed to determine the viability of LO2 cells treated with various concentrations of 3-MA for 24 h. (b) The level of the LDH released in LO2 cells treated with various concentrations of 3-MA for 24 h was measured using the LDH cytotoxicity assay kit. (c, d) LO2 cells were treated with 5 mM 3-MA for 24 h and stained with the LPO-specific dye BODIPY 581/591 C11, and LPO was detected by flow cytometry. (e, f) LO2 cells were treated with 5 mM 3-MA for 24 h and stained with Annexin V-FITC and PI, and then the cells that underwent apoptosis were quantified by flow cytometry. (g, h) Representative Western blots and quantitative densitometry analysis of the expression of the ACSL4 and LC3-II proteins in LO2 cells cultured with 3-MA for 24 h. (i, j) LO2 cells were treated with BSA, BSA+Rap (50 nM), PA (300 *μ*M), or PA (300 *μ*M)+Rap (50 nM) for 24 h. Representative Western blots and quantitative densitometry analysis of the expression of ACSL4 and LC3 proteins in LO2 cells. All data are presented as the mean ± Std Dev of at least three separate experiments; ns: nonsignificant; ⁣^∗^*p* < 0.05 and ⁣^∗∗^*p* < 0.01 between the indicated groups.

Ferroptosis is an autophagy-dependent type of cell death, and ACSL4, the initiator of ferroptosis, can undergo degradation through the autophagy-lysosomal pathway. We hypothesized that autophagy might modulate ferroptosis by influencing the level of the ACSL4 protein. To test this hypothesis, LO2 cells were treated with the autophagy inhibitor 3-methyladenine (3-MA). The results showed that 5 mM 3-MA decreased cell viability (Figure 7(a)), increased cytotoxicity (Figure 7(b)), and promoted the apoptosis of LO2 cells (Figures 7(e) and 7(f)). Using BODIPY 581/591 C11, we found that lipid ROS levels increased after treatment with 3-MA compared to that in the control group (Figures 7(c) and 7(d)). Moreover, the level of the ACSL4 protein was significantly higher in 3-MA-treated cells relative to that in the cells of the control group (Figures 7(g) and 7(h)). These results indicated that ferroptosis in LO2 cells was stimulated by the autophagy inhibitor 3-MA. In contrast, the level of expression of LC3-II was higher, but that of ACSL4 was lower in LO2 cells that were treated with the autophagy inducer rapamycin (Figures 7(i) and 7(j)). These findings suggested that autophagy may contribute to ferroptosis by regulating the expression of ACSL4.

## 4. Discussion

**Figure 8:**
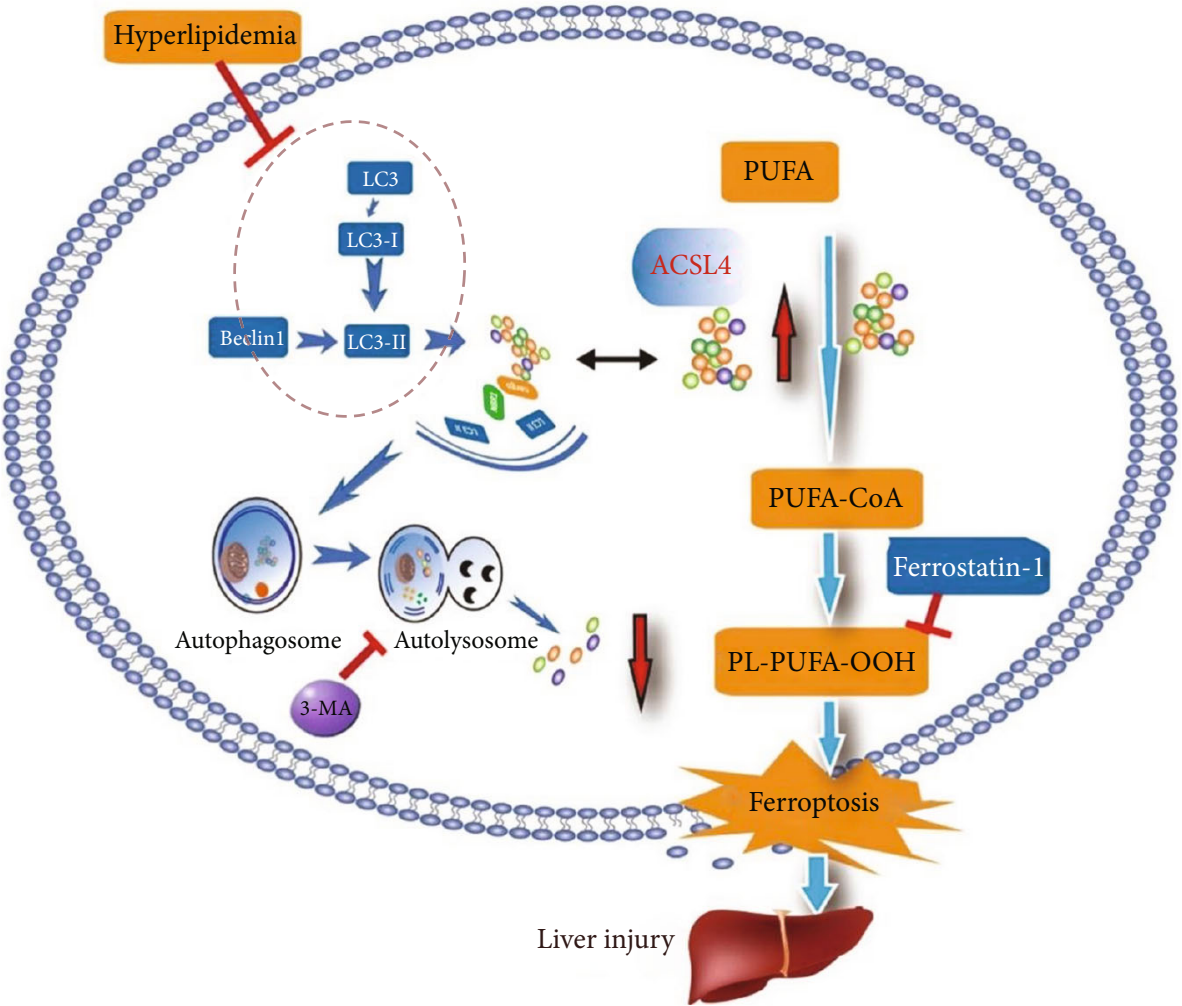
Proposed working model of autophagy in regulating ferroptosis via ACSL4 in diabetic liver injury. In diabetes, hyperlipidemia may induce an increase in the level of expression of ACSL4 by inhibiting autophagic flux, which can lead to the ferroptosis of hepatocytes, eventually resulting in diabetic liver injury.

Diabetic liver injury is a common complication of DM, but its exact etiology remains unknown. Ferroptosis is a type of regulated cell death mechanism associated with various diseases. Some studies have shown that an increase in ACSL4-induced ferroptosis is linked to the progression of diabetes, diabetic nephropathy [37, 38], and diabetes myocardial injury [39]. Previous studies have suggested the involvement of ferroptosis in diabetes-induced liver pathology [40], but whether ACSL4-mediated ferroptosis leads to diabetic liver injury is worth our further study. To address this gap, we investigated the mechanisms underlying the changes in ferroptosis in the liver of diabetic rats and PA-stimulated LO2 cells. We found an increase in ACSL4 levels and the subsequent induction of ferroptosis in the liver of diabetic rats and the PA-stimulated LO2 cells. Considering the prior theory that ferroptosis is a form of autophagy-dependent cell death [41], we found that ferroptotic changes were accompanied by the inhibition of autophagy both in vitro and in vivo. More importantly, ACSL4 was degraded via the autophagy-lysosome pathway. Further experiments showed that autophagy promoted ferroptosis by regulating the degradation of ACSL4, ultimately leading to diabetic liver injury (Figure 8). Future studies can use these findings as a theoretical basis for developing strategies for alleviating diabetic hepatic impairment by siACSL4 or by specifically inhibiting ferroptosis.

Many researchers have reported that high levels of free fatty acids in DM stimulate the liver to synthesize and release large quantities of TG and low-density lipoproteins [42]. This process results in the accumulation of high levels of fat in the liver, giving rise to lipotoxic metabolites, increasing the oxidative stress response in the liver, and ultimately exacerbating liver damage [43]. Similar to the findings of previous studies, we recorded high levels of lipids in the serum, high levels of hepatic enzymes, and lipid deposition in the livers of STZ-induced diabetic rats. These changes indicated that the diabetic rats suffered from hyperlipidemia and impaired liver function after 12 weeks of diabetes induction.

Hepatocellular death is a key contributor to the pathology of diabetic liver and the progression of liver damage. To determine whether ferroptosis plays a pivotal role in diabetic liver damage, we conducted several experiments and found that ferroptosis occurred in the liver of STZ-induced diabetic rats and the hepatocyte cell line LO2 stimulated with PA. High levels of Fe^2+^ in the serum and liver tissue of diabetic rats may contribute to the accumulation of iron-dependent ROS and the initiation of ferroptosis [44] in the diabetic liver. This finding is consistent with the very few researches that have linked ferroptosis to the progression of diabetic liver injury in STZ-induced diabetic mice [40] and db/db mice [45]. Diabetes is characterized by HG and hyperlipidemia. Our findings showed that the diabetic rats had high serum lipid profiles and lipid deposition. We recorded noticeable ferroptotic phenotypes, including LPO accumulation, increased cell death, and ferroptotic gene activation in LO2 cells treated with PA but not in cells treated with HG. Thus, these results suggested hyperlipidemia is a critical factor contributing to hepatocyte ferroptosis in diabetes. And to explore the effect of HG on ferroptosis warrants further investigation, possibly by adjusting intervention conditions or using different liver cell types. These findings suggested that besides undergoing conventional modes of cell death, such as apoptosis [46] and necrosis, diabetic hepatocytes also undergo ferroptosis. Our study provides a fresh mechanistic perspective on liver injury associated with diabetes.

ACSL4 is a critical determinant of the membrane lipid composition, and it sensitizes cells to ferroptosis, and increased ACSL4 has crucial contributions to diabetic complications [10]. However, research on the role of ACSL4 in ferroptosis within diabetic hepatocytes is limited. This study is the first to report increased ACSL4 expression in PA-treated LO2 cells and STZ-induced diabetic rat livers. Consistent with previous findings, we observed that ACSL4 upregulation promotes ferroptosis in diabetic hepatocytes. And ferroptosis inhibitor Fer-1 can reduce ACSL4 expression, thereby alleviating LPO and preventing PA-induced ferroptosis in LO2 cells. Additionally, siRNA-mediated knockdown of ACSL4 improved cell viability and mitigated LPO, confirming that ferroptosis is a key mode of cell death induced by high lipids in diabetes. Our study provided valuable insights into the mechanism of diabetic liver injury associated with ferroptosis. Targeting ACSL4 may be an effective strategy for the prevention and treatment of diabetic liver injury.

As ferroptosis is a type of autophagy-dependent programmed cell death, it requires an autophagy mechanism. Researchers have reported a close association between autophagy inhibition and the pathogenesis of DM [21] and its complications, including diabetic cardiomyopathy [47], diabetic nephropathy [48], and diabetic liver damage [49, 50]. In our study, the level of expression of autophagy-associated proteins decreased and that of p62 increased, indicating that autophagy was inhibited in the liver tissues of diabetic rats. These results were similar to those of previous studies that found that autophagy dysregulation contributes to diabetic liver injury [26, 27]. Since PA-induced ferroptosis in the above experiment, we used PA for subsequent experiments to mimic hyperlipidemia and record the changes related to autophagy in diabetes. The effect of PA on autophagy differed among cells under various conditions [47, 51–53]. Our findings showed that autophagic flux was inhibited in LO2 cells treated with PA, which was similar to the findings of other studies that reported that PA can suppress autophagy in neurons [54], cardiomyocytes [47], and LO2 cells [52, 53]. Overall, we found a concomitant occurrence of ferroptosis and suppression of autophagy in the liver of diabetic rats and PA-stimulated LO2 cells. Various autophagy-related mechanisms contribute to ferroptosis. Therefore, investigating whether autophagy regulates ACSL4 to mediate ferroptosis is a valuable area of research. We found that PA improved ACSL4 stability and hindered its degradation. And ACSL4 was predominantly degraded via the autophagy-lysosomal pathway, rather than the ubiquitin-proteasome pathway, in PA-treated LO2 cells. However, some studies have found that arachidonic acid can induce ACSL4 degradation through the ubiquitin-proteasomal pathway in hepatic cells [55]. These differences in degradation pathways might be due to the application of different cell lines and intervention modalities. The result of another study that ACSL4 is a substrate of chaperone-mediated autophagy also supported our opinion [56]. In addition, the autophagy inhibitor 3-MA decreased cell viability, increased LPO, and upregulated the ferroptosis marker ACSL4. In contrast, rapamycin-induced autophagy significantly reduced ACSL4 expression in LO2 cells. Therefore, our findings have indicated that autophagy-mediated ACSL4 degradation was associated with ACSL4 protein accumulation under those conditions. In our study, while we have observed concomitant upregulation of ACSL4 mRNA and protein levels, we consider that autophagy-mediated degradation of ACSL4 and its subsequent accumulation play a primary role in this process. We also observed that the upregulation of ACSL4 mRNA induced by PA may contribute to the increased abundance of ACSL4 protein. However, the underlying molecular mechanisms through which PA regulates ACSL4 transcription remain fully elucidated, highlighting a limitation of our current study. Possible reasons may include PA's role as a signaling molecule that activates specific transcription factors or pathways, such as the mTOR or ERK pathways, which are known to influence gene expression. Additionally, we will explore how PA may affect chromatin remodeling and recruitment of transcriptional coactivators to enhance ACSL4 transcription. Further studies are warranted to investigate the underlying molecular mechanisms by which PA regulates ACSL4 transcription. These findings further suggest that autophagy can regulate ferroptosis in LO2 cells through ACSL4. In summary, given the driving role of ACSL4 in ferroptosis, we elucidated a new mechanism by which autophagy can induce ferroptosis by regulating the degradation of ACSL4.

## 5. Conclusions

This study investigated the roles of ACSL4-mediated ferroptosis and autophagy in the mechanisms underlying diabetic liver injury, emphasizing their interconnection. We found that ferroptosis occurred in the livers of diabetic rats and in PA-treated LO2 cells, accompanied by the inhibition of autophagy. Our results also indicated that ACSL4 levels increased in diabetes and were degraded through the autophagy-lysosomal pathway. We elucidated a novel mechanism by which autophagy can mediate ferroptosis by regulating the expression of ACSL4 in diabetic hepatocytes, thereby contributing to diabetic liver damage. However, further investigation is required to determine whether the regulation of autophagy and ACSL4 can mitigate ferroptosis and reduce diabetic liver injury. In conclusion, our findings not only offer novel insights into the mechanisms and therapeutic targets for diabetic liver injury but also provide valuable clues for understanding the pathogenesis of ferroptosis and developing therapeutic strategies for other diabetic complications.

## Figures and Tables

**Table 1:** Body weight, blood glucose levels, and biochemical indicator levels in blood and liver of rats.

	**Con (** **n** = 4**)**	**DM (** **n** = 4**)**	**p**
Body weight (g)	494.00 ± 24.71	309.25 ± 29.48^∗∗^	≤ 0.001
Blood glucose (mmol/L)	7.10 ± 0.50	31.43 ± 1.84^∗∗^	≤ 0.001
ALT (U/L)	27.40 ± 4.90	108.30 ± 43.47^∗^	0.033
AST (U/L)	40.56 ± 4.90	117.78 ± 42.82^∗^	0.012
TG (mmol/L)	0.80 ± 0.11	3.08 ± 0.77^∗∗^	0.001
TC (mmol/L)	1.32 ± 0.12	2.77 ± 0.54^∗∗^	0.002
Serum MDA (nmol/mL)	6.13 ± 1.10	13.93 ± 4.76^∗∗^	0.002
Liver MDA (nmol/mg)	1.46 ± 0.24	3.16 ± 0.54^∗∗^	0.001
Serum Fe^2+^(*μ*mol/L)	20.81 ± 5.35	61.73 ± 23.31^∗^	0.014
Liver Fe^2+^(*μ*mol/g)	9.05 ± 2.50	18.51 ± 6.89^∗^	0.042
Serum SOD (U/mL)	658.05 ± 102.38	349.27 ± 68.74^∗∗^	0.002
Liver SOD (U/mg)	894.23 ± 97.68	320.74 ± 86.72^∗∗^	≤ 0.001

Abbreviations: ALT, alanine transaminase; AST, aspartate aminotransferase; MDA, malondialdehyde; SOD, superoxide dismutase; TC, total cholesterol; TG, triglyceride.

⁣^∗^* p* <0.05.

⁣^∗∗^* p* <0.01 versus Con.

## Data Availability

The data that support the findings of this study are available on request from the corresponding authors.

## Supporting Information

Supporting Information 1 Figure S1: Effects of HG and PA on the activity, toxicity, and death of LO2 cells. (a, b) The effect of various concentrations of glucose or mannitol on the viability of LO2 cells cultured for 48 or 72 h was evaluated. Cell viability was assessed by conducting a CCK-8 assay. (c) The level of LDH released was measured using the LDH cytotoxicity assay kit in LO2 cells treated with various concentrations of glucose or mannitol for 72 h. (d, e) The LO2 cells were treated with 33 mM HG for 72 h and stained with Annexin V-FITC and PI, and then the cells that underwent apoptosis were quantified by flow cytometry. (f) A CCK-8 assay was performed to determine the viability of LO2 cells treated with various concentrations of PA or BSA for 24 h. All data are presented as the mean ± Std Dev of at least three independent experiments; ns: nonsignificant; ⁣^∗^*p* < 0.05, ⁣^∗∗^*p* < 0.01, and ⁣^∗∗∗^*p* < 0.001 between the indicated groups.

Supporting Information 2 Figure S2: ACSL4 overexpression induces ferroptosis in LO2 cells. (a) qPCR analysis of ACSL4 mRNA expression in control and ACSL4 overexpression LO2 cells. (b) Immunoblotting analysis of ACSL4 protein expression in control and ACSL4 overexpression LO2 cells. (c) Rate of cell death of LO2 cells transfected with control and ACSL4 overexpression plasmids. (d) Cell viability of LO2 cells transfected with control and ACSL4 overexpression plasmids. (e) Rate of LPO of LO2 cells transfected with control and ACSL4 overexpression plasmids. Bars represent the mean ± Std Dev; ⁣^∗∗^*p* < 0.01 between the indicated groups.

Supporting Information 3 Figure S3: The structure of the ACSL4 overexpression plasmid.

Supporting Information 4 Figure S4: High expression of ACSL4 in the liver of db/db mice. Representative images and quantitative analysis of ACSL4 in the liver, as determined by IHC; scale bar: 50 *μ*m. All data are presented as the mean ± Std Dev; *n* = 4 per group; ⁣^∗∗∗^*p* < 0.001 versus Con.
