# Supplementary figures and images for: Autophagy Regulates Ferroptosis-Mediated Diabetic Liver Injury by Modulating the Degradation of ACSL4
Source: J Diabetes Res. 2024 Dec 24;2024:7146054. doi: 10.1155/jdr/7146054 (PMC11688137; doi:10.1155/jdr/7146054)

(a)

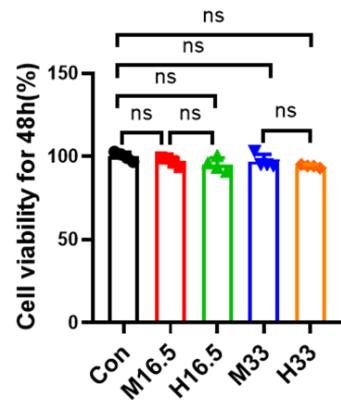

(b)

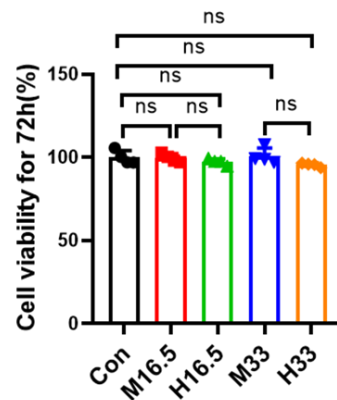

(c)

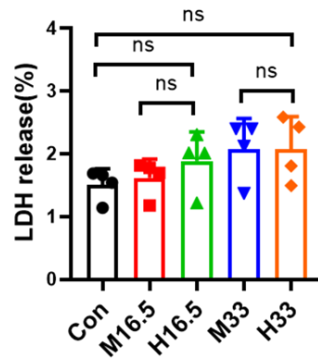

(d)

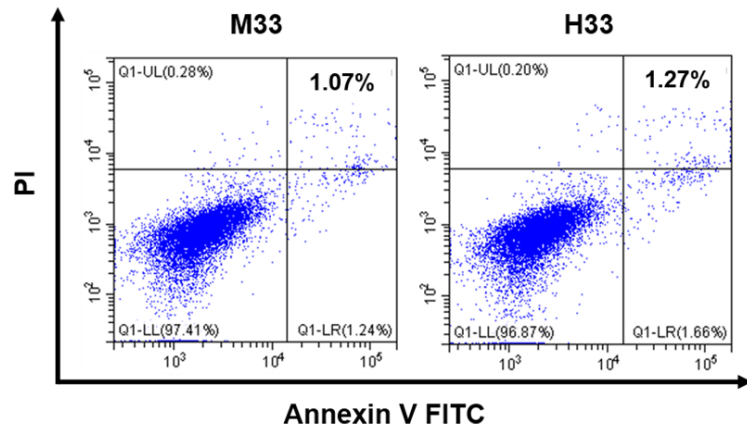

(e)

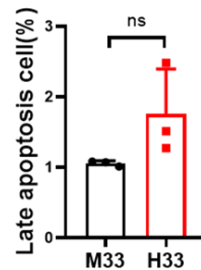

(f)

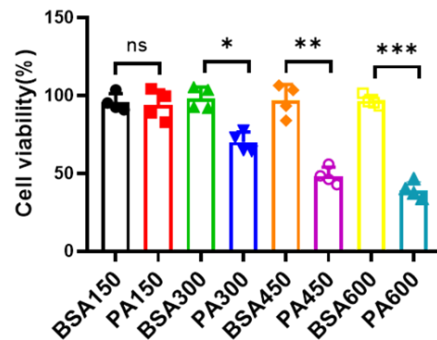

Supplement: Supporting Information 1 — Figure S1: Effects of HG and PA on the activity, toxicity, and death of LO2 cells. (a, b) The effect of various concentrations of glucose or mannitol on the viability of LO2 cells cultured for 48 or 72 h was evaluated. Cell viability was assessed by conducting a CCK-8 assay. (c) The level of LDH released was measured using the LDH cytotoxicity assay kit in LO2 cells treated with various concentrations of glucose or mannitol for 72 h. (d, e) The LO2 cells were treated with 33 mM HG for 72 h and stained with Annexin V-FITC and PI, and then the cells that underwent apoptosis were quantified by flow cytometry. (f) A CCK-8 assay was performed to determine the viability of LO2 cells treated with various concentrations of PA or BSA for 24 h. All data are presented as the mean ± Std Dev of at least three independent experiments; ns: nonsignificant; ⁣∗p < 0.05, ⁣∗∗p < 0.01, and ⁣∗∗∗p < 0.001 between the indicated groups. [file 7146054.f1.pdf]

(a)

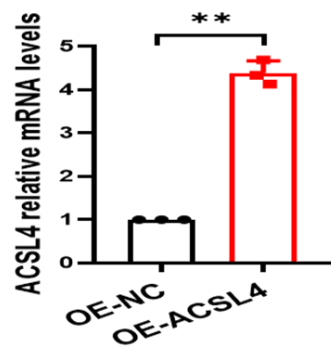

(b)

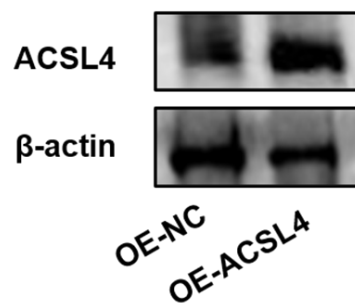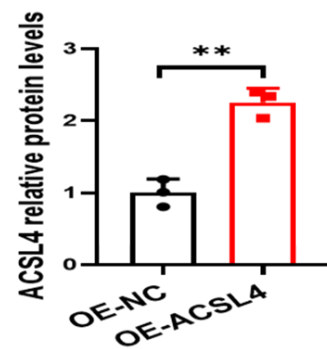

(c)

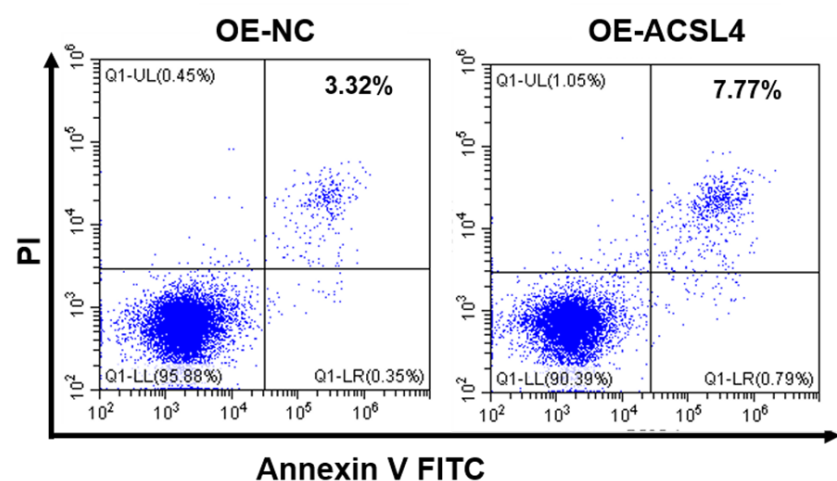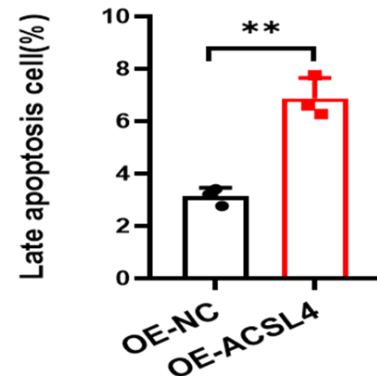

(d)

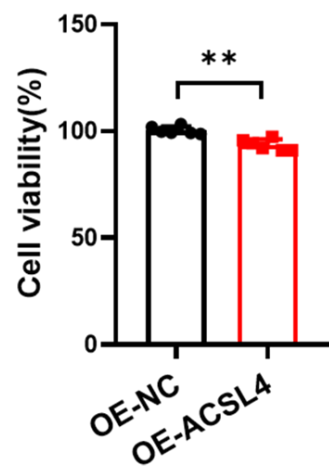

(e)

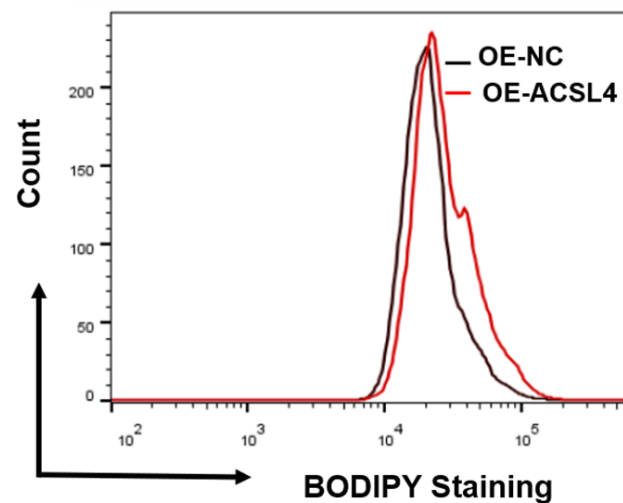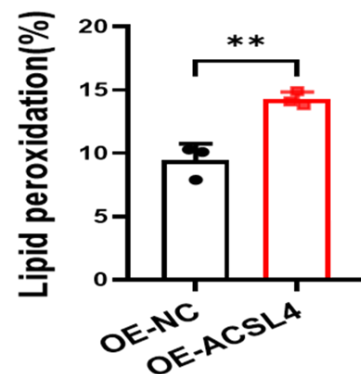

Supplement: Supporting Information 2 — Figure S2: ACSL4 overexpression induces ferroptosis in LO2 cells. (a) qPCR analysis of ACSL4 mRNA expression in control and ACSL4 overexpression LO2 cells. (b) Immunoblotting analysis of ACSL4 protein expression in control and ACSL4 overexpression LO2 cells. (c) Rate of cell death of LO2 cells transfected with control and ACSL4 overexpression plasmids. (d) Cell viability of LO2 cells transfected with control and ACSL4 overexpression plasmids. (e) Rate of LPO of LO2 cells transfected with control and ACSL4 overexpression plasmids. Bars represent the mean ± Std Dev; ⁣∗∗p < 0.01 between the indicated groups. [file 7146054.f2.pdf]

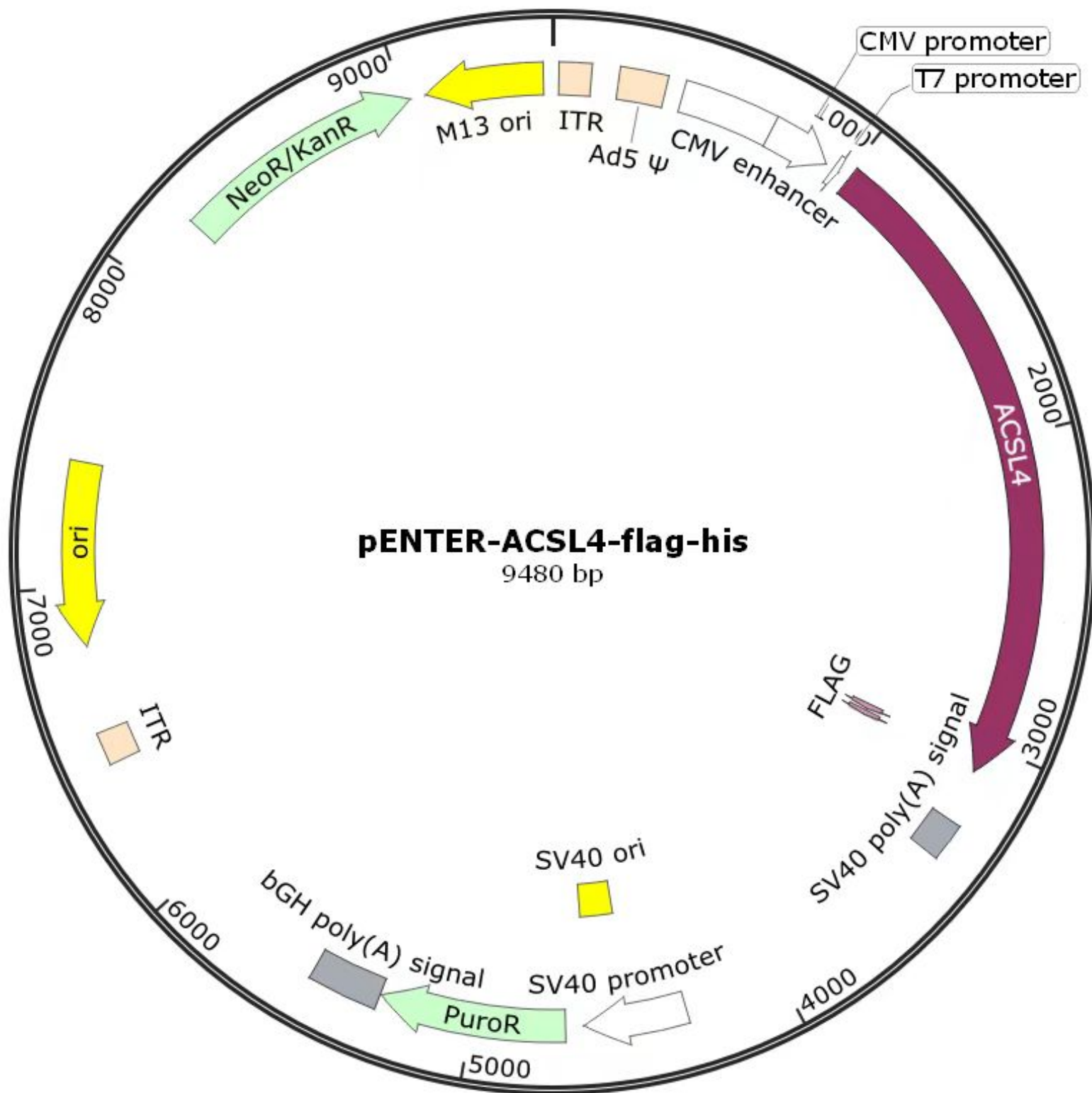

Supplement: Supporting Information 3 — Figure S3: The structure of the ACSL4 overexpression plasmid. [file 7146054.f3.pdf]

ACSL4

WT

db/db

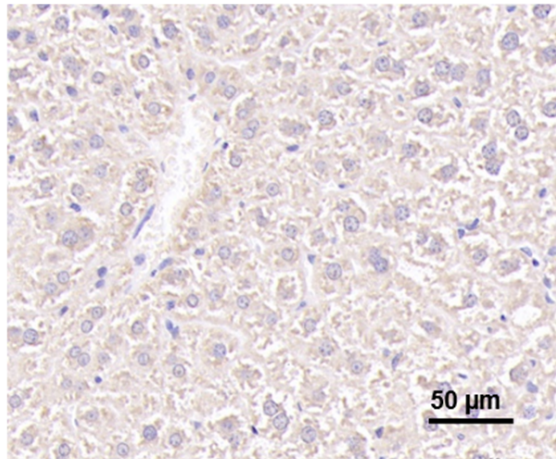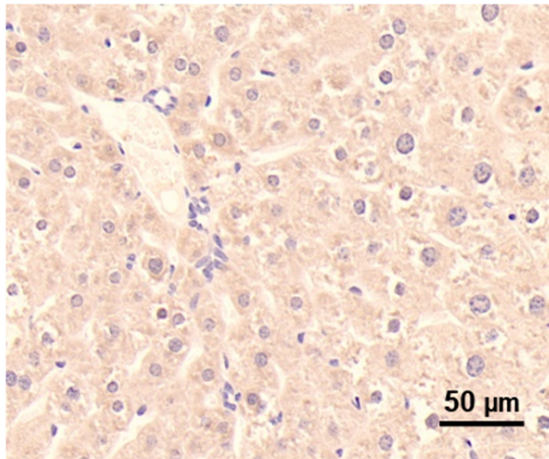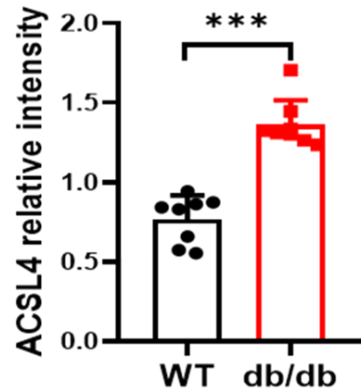

Supplement: Supporting Information 4 — Figure S4: High expression of ACSL4 in the liver of db/db mice. Representative images and quantitative analysis of ACSL4 in the liver, as determined by IHC; scale bar: 50 μm. All data are presented as the mean ± Std Dev; n = 4 per group; ⁣∗∗∗p < 0.001 versus Con. [file 7146054.f4.pdf]
